# Striatal *FoxP2* Is Actively Regulated during Songbird Sensorimotor Learning

**DOI:** 10.1371/journal.pone.0008548

**Published:** 2010-01-06

**Authors:** Ikuko Teramitsu, Amy Poopatanapong, Salvatore Torrisi, Stephanie A. White

**Affiliations:** 1 Interdepartmental Program in Molecular, Cellular and Integrative Physiology, University of California Los Angeles, Los Angeles, California, United States of America; 2 Interdepartmental Program in Neuroscience, University of California Los Angeles, Los Angeles, California, United States of America; 3 Department of Physiological Science, University of California Los Angeles, Los Angeles, California, United States of America; Max-Planck-Institut für Neurobiologie, Germany

## Abstract

**Background:**

Mutations in the FOXP2 transcription factor lead to language disorders with developmental onset. Accompanying structural abnormalities in cortico-striatal circuitry indicate that at least a portion of the behavioral phenotype is due to organizational deficits. We previously found parallel *FoxP2* expression patterns in human and songbird cortico/pallio-striatal circuits important for learned vocalizations, suggesting that FoxP2's function in birdsong may generalize to speech.

**Methodology/Principal Findings:**

We used zebra finches to address the question of whether FoxP2 is additionally important in the post-organizational function of these circuits. In both humans and songbirds, vocal learning depends on auditory guidance to achieve and maintain optimal vocal output. We tested whether deafening prior to or during the sensorimotor phase of song learning disrupted *FoxP2* expression in song circuitry. As expected, the songs of deafened juveniles were abnormal, however basal *FoxP2* levels were unaffected. In contrast, when hearing or deaf juveniles sang for two hours in the morning, *FoxP2* was acutely down-regulated in the striatal song nucleus, area X. The extent of down-regulation was similar between hearing and deaf birds. Interestingly, levels of *FoxP2* and singing were correlated only in hearing birds.

**Conclusions/Significance:**

Hearing appears to link *FoxP2* levels to the amount of vocal practice. As juvenile birds spent more time practicing than did adults, their *FoxP2* levels are likely to be low more often. Behaviorally-driven reductions in the mRNA encoding this transcription factor could ultimately affect downstream molecules that function in vocal exploration, especially during sensorimotor learning.

## Introduction

Forkhead box (FOX) genes encode a family of transcription factors that play regulatory roles during development [1], [2]. FOXP2, a member of this family, is the first gene to be directly linked to human language [3]–[5]. Humans with FOXP2 mutations exhibit deficits in the coordination of sequential orofacial movements, resulting in impaired speech (developmental verbal dyspraxia) [6]. This core deficit is accompanied by additional impairments in receptive linguistic skills and abnormal activation of cortico-basal ganglion regions used in verbal communication [7]. Together, these observations implicate FOXP2 in the organization of neural structures necessary for speech and language.

Birdsong shares key features with speech: it is learned during development, actively maintained in adulthood, requires hearing and relies on pallio-striatal circuits [8], [9]. The neuroanatomical structures that subserve song learning and production, known as song nuclei, are well-characterized [10]–[13]. Songbirds thus provide an important model for the study of neural mechanisms underlying vocal learning. *FoxP2* is expressed in the striatum of human embryos and of 1 day post hatch (1d) zebra finches [14]. *FoxP2* levels appear to increase in the song nucleus, area X, of developing zebra finches at 35 and 50d [15], followed by an increase in area X volume and the number of new neurons expressing FoxP2 protein at 50 and 75d [16]. Area X is the region of the songbird basal ganglia dedicated to song [17], and contains neuronal phenotypes, including medium spiny neurons, similar to those in mammalian basal ganglia [18], [19]. These observations, coupled with the anatomical abnormalities of humans bearing FOXP2 mutations [20], support a role for FoxP2 in the development of neural structures that subserve vocal learning.

In addition to this organizational role, FoxP2 may have post-organizational function(s) in learned vocalizations as its mRNA and protein are rapidly down-regulated specifically in area X when adult zebra finches practice their songs outside the context of courtship (i.e. sing undirected songs) [21], [22]. This idea is supported by the known role of the anterior forebrain pathway, which includes area X, in enabling song modification during development [23], [24] and throughout life [25]. Here, we used zebra finches (*Taeniopygia guttata*) to investigate the role of FoxP2 in song learning. In this species, young males memorize the song of adult male tutors, and then practice their songs during a phase known as ‘sensorimotor learning’ which spans ∼30–100 d [26], [27]. The learned songs are actively maintained in relatively stable form throughout adulthood [28], [29] when the sizes of song nuclei are also relatively stable [30]. Thus, the *FoxP2* down-regulation observed in area X of adult zebra finches cannot be due to developing new songs or to significant restructuring of song circuitry. Instead, acute down-regulation may reflect an on-line function for FoxP2 during singing. This function could be to help stabilize mature song. If so, then down-regulation might be lessened or absent in juveniles. Alternatively, acute *FoxP2* down-regulation in adults might enable subtle adjustments involved in song maintenance. In this case, reduction of *FoxP2* might be similar or greater in juveniles, as they make greater modifications to their songs during learning. In either case, the on-line regulation could be associated with motor control and/or auditory feedback of song.

To probe these possibilities, we first examined basal levels of *FoxP2* in area X of non-singing hearing or deafened birds during sensorimotor learning. Our findings suggest that basal *FoxP2* levels are associated with structural growth of area X and are not affected by deafening. We then tested for acute down-regulation of *FoxP2* in area X of 75 d birds as a function of singing for two hours in the morning. We found that when juveniles sang, *FoxP2* levels in area X declined, similar to what we previously reported for adults. Therefore, this regulation is more likely related to song adjustment than to song stability. Interestingly, singing decreased *FoxP2* in both hearing and deafened birds, however, levels were only correlated with the amount of singing in hearing birds. Here, we report this evidence for both motor and auditory regulation of *FoxP2*.

## Materials and Methods

### Animals and Tissues

All animal husbandry and experimental procedures were in accordance with NIH guidelines for experiments involving vertebrate animals and approved by the University of California, Los Angeles Institutional Animal Care and Use Committee. Birds were fed seed and calcium-enriched (Calciboost, The Birdcare Company, Gloucestershire, UK) water *ad libitum*, provided with weekly nutritional and environmental supplements (hard-boiled chicken egg, fresh carrots and komatsuma, millet sprays, bathing water) and kept on a 12.5 hr-light/11.5 hr-dark cycle. Forty-three male zebra finches raised in our breeding colony were used for measurements of song and striatal *FoxP2* mRNA levels. By convention, when referring to mRNA, *FoxP2* is italicized to distinguish it from FoxP2 protein [1]. An additional 14 birds were examined solely for daily patterns of singing.

Songs were recorded when birds were singly housed in sound attenuation chambers (Acoustic Systems; Austin, TX); conditions under which all singing is, by definition, undirected [31]. *FoxP2* levels in area X were examined in birds as a function of their age (50, 65 or 75 d), behavioral state (non-singing or singing) and auditory capacity (hearing or deaf). Throughout the text, these groups are indicated by names and acronyms as follows: 50 d non-singing hearing (**50NS-H**; n = 6), 50 d non-singing deaf (**50NS-D**; n = 4), 65d non-singing hearing (**65NS-H**; n = 3), 65d non-singing deaf (**65NS-D**; n = 3), 75d non-singing hearing (**75NS-H**; n = 3) or 75d non-singing deaf (**75NS-D**; n = 4). In addition to these non-singing groups, two singing groups were tested at 75d: singing hearing (**75S-H**; n = 7) and singing deaf (**75S-D**; n = 10). An additional three hearing 75d birds were also tested (see below for rationale). Birds were killed via rapid decapitation, and brains were quickly extracted, frozen on liquid nitrogen and stored at −80°C until use.

### Deafening

Juvenile male zebra finches (n = 4 at 25d or n = 17 at 35d) were deafened by bilateral removal of the cochlea as described in Konishi (1965) [32]. Briefly, birds were anesthetized with barbiturate anesthetic, equithesin (intrapectorally: 0.85 g chloral hydrate/4.2 ml pentobarbital/0.42 g MgSO_4_/6.92 ml propylene glycol/1.78 ml 100% ethanol to a total volume of 20 ml with water, then filtered) and secured on a rotary table. Under a dissection microscope (OPMI pico, Carl Zeiss Meditec, Inc., Dublin, CA), a small area of skin as well as the tympanic membrane overlaying the middle ear cavity was removed using iridectomy scissors, followed by the removal of the columella, allowing visualization of the cochlea. A small hook made of tungsten fiber was used to extract the cochlea. Removal of an unbroken cochlea indicated the initial success of the surgery, which was later confirmed by song analysis (see below). Following surgery, Neosporin® (Pfizer, Morris Plains, NJ) was applied to each ear, and birds were monitored on a homeothermic blanket (Harvard apparatus Ltd., Edenbridge, UK) until recovery from anesthesia when they were returned to their parents in breeding cages. Antibiotic (Baytril, Bayer Animal Health, Shawnee Mission, KS; prior to the Federal Drug Administration ban on the product) was added to the drinking water for 10 days.

### Sham Surgeries

Three additional birds underwent sham operations to control for any potential effects of the surgical procedure itself. Sham operations consisted of the same anesthetic protocol and skin removal as the deafened birds above, but without damage to the tympanic membrane or cochlear extraction. Data from 3 birds that were sham-operated at 25d were compared to that from 3 birds that did not receive sham treatment (untreated). These sham operated or untreated birds were examined at 50d, and their non-singing ‘basal’ *FoxP2* levels in area X were compared using *in situ* hybridization analyses with two distinct probes for *FoxP2* as described in Teramitsu & White (2006) and below. Using photomicrographs, the pixel intensity of the hybridization signal in area X relative to that in the outlying striatum of the same hemi-coronal section was calculated as a ratio. Multiple sections per bird were analyzed and a per bird average was computed and used for statistical comparison (see below for more details). As expected, no differences in area X *FoxP2* levels were observed between sham operated and untreated birds, indicating that the surgery itself had no effect (mean±SEM levels in area X relative to levels in the outlying striatum, Sham vs. Untreated – 3′ probe: 1.05±0.06 vs. 1.09±0.03, *p* = 0.38; mid-probe: 1.05±0.04 vs. 1.11±0.04, *p* = 0.19). Therefore, the results from sham operated and untreated birds are pooled below, and these birds are henceforth referred to as 50NS-H group.

### Basal FoxP2 Levels in Non-Singing Birds

**Figure 1:**
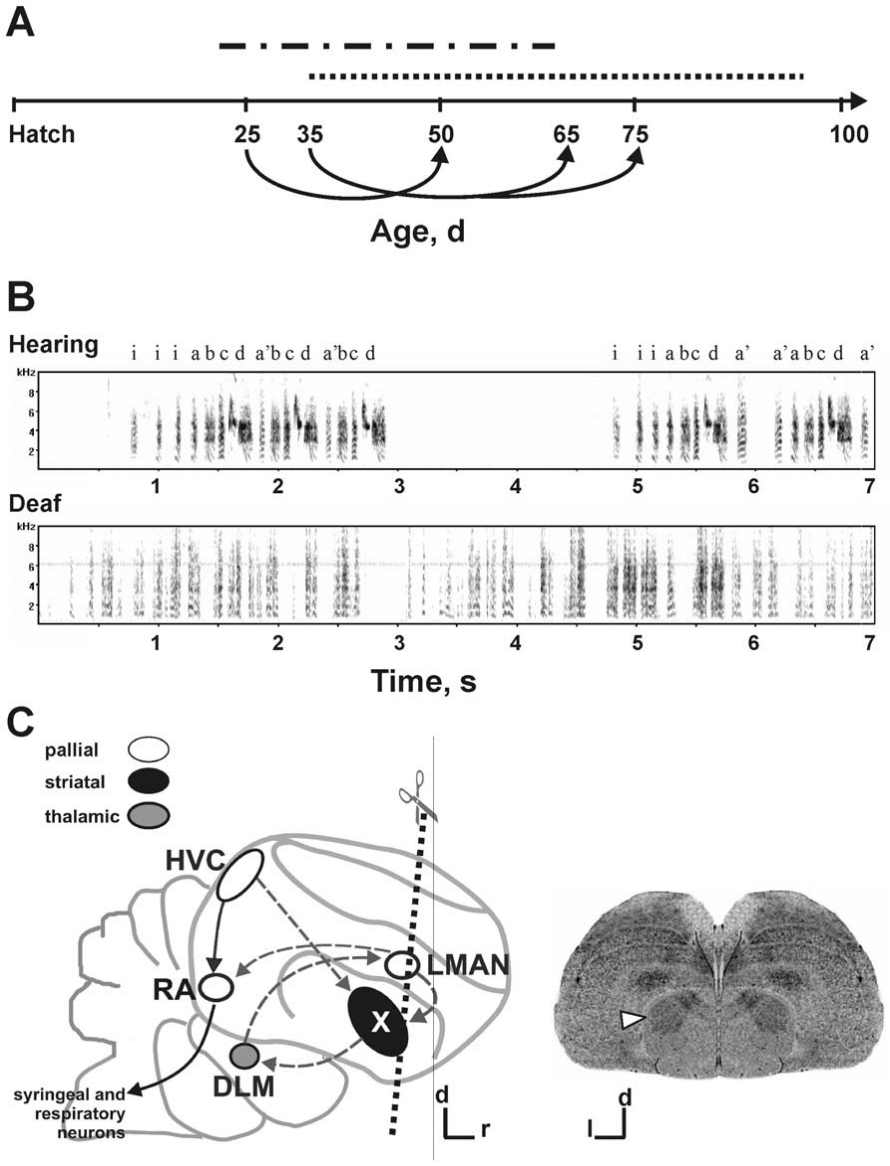
Deafening at 35d causes abnormal song development. **A**) Time line for experiments conducted during song learning which ends at ∼90d. One group of birds was either untreated, sham-operated or deafened at 25d, during sensory acquisition (dashed and dotted line) and prior to the onset of sensorimotor learning (dotted line). Their *FoxP*2 levels were measured at 50d. Another group of birds was either untreated or deafened at 35d, the onset of sensorimotor learning, and their *FoxP2* levels measured at either 65 or 75d. **B**) Exemplar spectrograms of a 75d hearing (top) and a deaf (bottom) bird. Although yet immature, the 75d hearing bird's song shows structures typical of zebra finch songs including introductory notes (i) and repeated motifs, which are composed of 4–7 easily identified syllables (a or a' – d). In contrast, songs of 75d deafened males were disrupted, and motifs were not identifiable. Signal at ∼6.5 kHz represents background noise. **C**) Left - schematic of major nuclei of the song circuit indicates the plane of section used to examine *FoxP2* levels in area X (arrowhead in the Nissl stain; right). Abbreviations: d – dorsal, DLM – medial portion of the dorsolateral nucleus of the anterior thalamus, HVC – acronym used as a proper name, l – lateral, LMAN – lateral magnocellular nucleus of the anterior nidopallium, r – rostral, RA – robust nucleus of the arcopallium, X – area X of the medial striatum. Axis lines underneath the Nissl section (right) indicate 1mm.

For analysis of *FoxP2* levels in hearing or deafened juvenile birds that did not sing on the day of the experiment, a total of 23 juvenile male zebra finches were used. Birds were monitored in the morning during the 20 minutes from light onset to decapitation to ensure that no singing occurred. To examine the effect of development on *FoxP2* expression, 50, 65 and 75d birds were used. As mentioned above, an additional three hearing 75d birds were used in order to facilitate comparison between our measurements and those obtained by another study (Haesler et al., 2004; see Discussion) [15]. This latter group was killed in the morning immediately at light onset rather than within 20 minutes after. Further, brains were sectioned in the sagittal, rather than the coronal, plane. Finally, the region of the outlying striatum that was measured was matched to the region used by Haesler et al. (2004). To examine the effect of auditory deprivation on *FoxP2* expression, a cohort of the birds were deafened at either 25 or 35d (Fig. 1A). Birds that were deafened at 25d were examined at 50d and compared to age-matched hearing birds. Birds that were deafened 35d were examined at 65 or 75d, and compared with age-matched hearing birds (Fig. 1A).

### FoxP2 Levels in Singing Birds

To test the effect of singing on *FoxP2* expression during sensorimotor learning in the presence or absence of auditory feedback, juvenile males were deafened at 35d. Experiments were conducted on these deafened birds and on age-matched hearing birds at 75d, in the morning between 8 AM and noon to minimize circadian variability. Following light onset, the singly housed deafened or hearing males were allowed to sing for 2 hr and then killed for measurement of *FoxP2* levels. Digital sound recordings were made using the Song Acquisition Program described in Livingston et al. (2000) [33]. The acoustic structure of birdsong is typically described as being composed of bouts, phrases, motifs, syllables, and notes [34]. Notes are the smallest unit, combining together to form syllables. Syllables are separated from one another by silent intervals. Two or more syllables may group together to form a phrase. A motif is a sequence of notes and/or syllables that are repeated in a stereotyped order. One or more motifs or phrases followed by a second or more of silence comprises a bout of song [35]. In this study, the number of motifs sung by each bird in the hearing group was counted. Because the songs of deafened birds lacked identifiable motif structures, the amount of time spent singing was also measured for both deafened and hearing groups. Silent periods longer than a second were regarded as bout intervals and were not included in the song measurement. For hearing birds, Sound Analysis Pro 1.04 software [36] was used to determine the degree of acoustic variability between syllables. Ten motifs per bird were analyzed for the within-syllable variability via 45 pair-wise comparisons of the acoustic features using the local similarity measure [37]. Resultant scores per syllable were then averaged for each bird.

### *In Situ* Hybridization Analyses

To measure *FoxP2* levels in area X of juvenile males, *in situ* hybridizations were performed following the methods of Teramitsu et al. (2004) [14]. As reported in that study, there are two major splice variants for the coding sequence of zebra finch *FoxP2*. In addition to the full-length form, a truncated variant lacks the forkhead DNA-binding domain but codes for an additional ten amino acids not present in the full-length form (GenBank DQ285023), similar to the so-called +10 form found in humans [38]. Hence, different hybridizing sequences were chosen to create two probes, one to the middle region of the coding sequence, which we refer to as the ‘mid-probe’, and the other to the 3′ end of the coding sequence, referred to as the ‘3’-probe’. The former can detect both of these *FoxP2* variants whereas the latter will only hybridize to the full-length variant. Of note, because the probes had slightly different specific activities and lengths [14], brain sections that were hybridized with the 3′-probe were exposed to separate films from those hybridized to the mid-probe. This avoided saturation of signals by the stronger probe. Consequently, comparisons of signal intensity between probes are not warranted.

*FoxP2* expression in area X of multiple coronal or sagittal brain sections was quantified from digitized images of film autoradiograms using Adobe Photoshop 7.0 (Adobe Systems Inc. San Jose, CA) as previously detailed [14]. Briefly, the background was subtracted from each image, and then the ‘histogram’ tool was used to measure the optical density (OD) values in area X or surrounding medial striatum (striatum mediale; StM). Respective areas for measurements were selected in an unbiased manner by using adjacent Nissl-stained sections precisely overlaid on the film images. For coronal sections, OD measurements were obtained from ∼13 hemi-sections per bird (i.e. both right and left hemispheres, if suitable for quantification, were analyzed) spanning the rostro-caudal extent of area X. A similar number of sagittal sections were used spanning the medio-lateral extent of area X. These measurements were averaged to provide a single value per region per bird. OD values from area X were normalized to those from adjacent StM. Thus, ratios of 1.0 indicate that expression levels in the two regions are comparable. Following this analysis, Feenders and colleagues found that gene expression levels in outlying striatum can vary as a function of behavior (e.g. hopping and flying [39]). Thus, in cases where we observed singing-driven regulation of *FoxP2* in area X using outlying striatum as a control tissue for normalization, we additionally measured *FoxP2* levels in nidopallial regions of the same section (outside of LMAN) and used these for normalization.

### Duration of Singing As a Function of Age and Breeding State

To determine how much time juvenile birds spent singing relative to adults, 75d or >120d males from our aviary and >120d pair-bonded males from breeding cages were placed individually in sound attenuation chambers for 5 consecutive days and their songs were recorded during this time. The songs of all subjects were sufficiently mature such that it was possible to identify each bird's motif, i.e. the kernel of acoustic structure defined by repeated sequences of syllables. For all ages, the number of motifs that each bird sang throughout the entire day on days 2–5 was manually counted using Audacity (v1.3). The circadian pattern of singing was noted by binning into 5 2.5-hour segments, beginning at lights-on (07:30 AM) and ending at lights-off (20:00 PM).

### Statistics

Non-parametric methods were used because the data did not conform to parametric assumptions. The effect of auditory deprivation on *FoxP2* expression was analyzed using the Kruskal-Wallis test for more than two groups, and the test statistic (*H*) with degrees of freedom (df) are reported in the relevant figure legends, or in the text in those cases where there is no figure. Mann-Whitney tests were used for comparison of two groups. Two-tailed significance was set at *p*<0.05, as no *a priori* hypothesis about the direction of any change in *FoxP2* levels between deaf and hearing animals was made. Means±SEMs are reported. Spearman rank tests were used to assess the relationship between amount of singing and *FoxP2* expression levels examined in area X.

## Results

### Deafening at the Onset of Sensorimotor Learning Disrupts Song Development, but Does Not Affect Basal FoxP2 Levels

**Figure 2:**
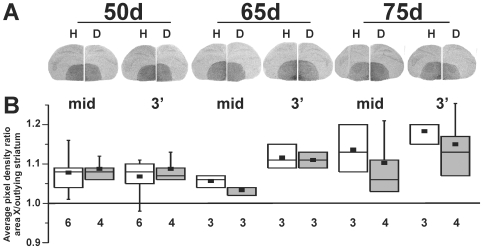
Basal *FoxP2* levels are similar between hearing and deafened juveniles. **A**) Exemplar hemi-coronal sections show *FoxP2* signals detected with either the mid or 3′-probe at 50, 65 and 75d in hearing (H; left hemi-sections) or deaf (D; right hemi-sections) birds. **B**) Quantification of pixel density within area X, normalized to values of the outlying striatum, reveals stable expression regardless of age or hearing condition. With each probe, at each age, and in each condition (white boxes = hearing, shaded boxes = deaf), values exceed unity (1.0), indicating slightly higher expression within area X. No differences were detected with either probe (Mean±SEM: H-NS vs. D-NS – 3′-probe: 50d, 1.07±0.02 vs. 1.09±0.02; 65d, 1.12±0.03 vs. 1.11±0.02; 75d, 1.18±0.02 vs. 1.15±0.07. Kruskal-Wallis *H* = 10.7, df = 5, *p* = 0.06; mid-probe: 50d, 1.08±0.02 vs 1.09±0.01; 65d, 1.06±0.01 vs. 1.03±0.01; 75d, 1.14±0.03 vs. 1.10±0.04. Kruskal-Wallis *H* = 8.7 *p* = 0.12). ‘Box and whiskers’ plots show the median (line), average (filled small rectangle), 25^th^ and 75^th^ percentiles (box) and 5^th^ and 95^th^ percent confidence intervals (whiskers) for each group. The number of birds per group is indicated beneath. For each bird, multiple sections were analyzed, then averaged, to produce a single metric per bird.

To confirm that song development was disrupted by deafening [32], [40], 75d hearing and deafened groups were recorded. The songs of the hearing males were well-structured with each motif composed of 4–7 readily identified syllables. In contrast, the songs of deafened males were highly disrupted, consisting of a series of amorphous syllables (Fig. 1B). No motif structures were reliably identified in any of the deafened birds. Although chronic auditory deprivation during sensorimotor learning produced abnormal songs, it did not alter basal *FoxP2* expression levels. In non-singing birds, *FoxP2* levels in area X were similar between hearing and deafened groups at 50, 65 and 75d (*p*>0.05 at each age, with either probe; Fig. 2).

Interestingly, levels of the full length *FoxP2* mRNA slightly increased over development (hearing and deafened birds pooled – 3′–probe: 50d, 1.08±0.01 vs. 65d, 1.11±0.01 vs. 75d, 1.16±0.02, *H* = 9.8, df = 2, *p*<0.01). In slight contrast, *FoxP2* levels detected by the mid-probe, designed to recognize both long and truncated forms of the molecule, exhibited a transient decrease at 65d followed by an increase at 75d (*H* = 6.8, df = 2, *p*<0.05). Given that the full length form shows a gradual, consistent rise across these ages, we interpret these data from the mid-probe as a dip in expression of the truncated form at 65d. The developmental changes in *FoxP2* expression levels shown here contrast slightly with a trend reported by Haesler et al. (2004) [15]. In that study, ratios of *FoxP2* levels within area X increased relative to outlying striatum from 15 to 50d but then appeared to return to 15d ratios at 75d. We were unable to replicate the reported return of basal area X *FoxP2* levels to at, or below, those of outlying striatum at 75d despite testing an additional three birds using methods more similar to that study (see Methods).

### FoxP2 Is Acutely Down-Regulated in Area X When 75d Juveniles Sing Undirected Songs

**Figure 3:**
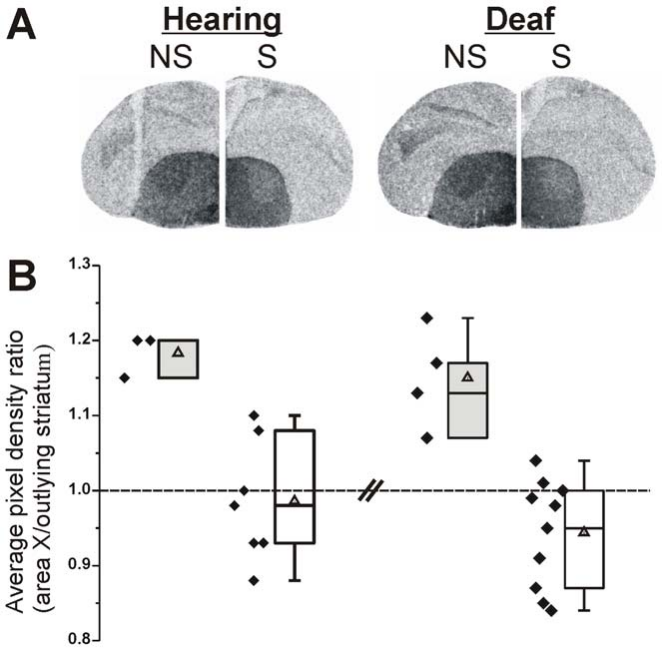
Singing down-regulates FoxP2 in both hearing and deaf juveniles. **A**) Representative sections show Fo*xP2* signals detected with the 3′-probe in hearing and deaf 75d birds. Signals within area X appear slightly stronger than in the surrounding striatum in the non-singer (NS), whereas they appear lower in area X of the singer (S). **B**) Quantification of the pixel intensity within area X is normalized to that of the outlying striatum. In both hearing (n = 7) and deaf (n = 10) birds, area X *FoxP2* levels are higher in the non-singing group (gray boxes) relative to the singing group (white boxes). Mean±SEM for hearing birds: 75NS-H vs. 75S-H: 1.18±0.02 vs. 0.99±0.04, Mann-Whitney *U* = 5.7, *p*<0.02. Mean±SEM for deaf birds: 75NS-D vs. 75S-D: 1.15±0.03 vs. 0.94±0.02, Mann-Whitney *U* = 8, *p*<0.005. ‘Box and whiskers’ plots show the median (line), average (triangle), 25^th^ and 75^th^ percentiles (box) and 5^th^ and 95^th^ percent confidence intervals (whiskers) for each group. Individual values are plotted to the left.

To investigate whether *FoxP2* in juvenile birds exhibits behavioral regulation similar to adults [21], we allowed 75d hearing birds to sing for 2 hours and examined the *FoxP2* levels in area X. We found that *FoxP2* in juveniles is also acutely down-regulated by singing (3′-probe, *p*<0.02; Fig. 3). Similar results were obtained with the mid-probe (data not shown). Because motor-driven gene expression can occur outside of area X for non-singing behaviors [39], we additionally measured *FoxP2* expression levels in a nidopallial region (outside of LMAN) on the same section and used these values for normalization. Akin to the prior analysis, *FoxP2* levels in area X were down-regulated by singing when nidopallial areas were used for normalization (3′-probe, *p*<0.02).

**Figure 4:**
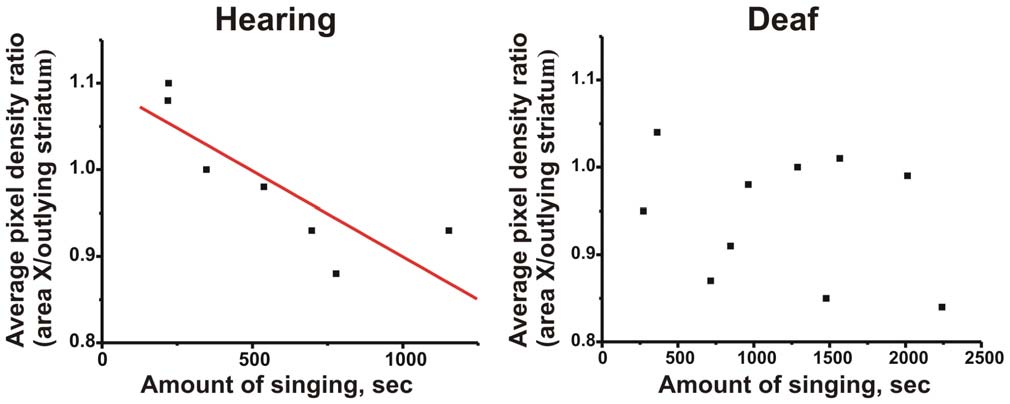
Hearing links amount of singing with FoxP2 levels. The amount of time that 75d birds spent singing (x axis) and area X *FoxP2* levels measured using the mid-probe (y axis) are correlated in hearing (left; Spearman Rho = −0.86, R^2^ = 0.69; *p*<0.02), but not in deaf (right; Spearman Rho = −0.19, R^2^ = 0.04; *p* = 0.60), juveniles.

The extent of *FoxP2* down-regulation was correlated with both the amount of time spent singing (Spearman Rho *p*<0.02; Fig. 4 left) and with the number of motifs sung (*p*<0.02). As expected, the songs of these juvenile birds were less stable than those of the adult males we previously studied (juveniles vs. adults: mean accuracy of syllables (75d range = 76–82%, average = 79%±0.9 vs. adult range = 82–90%, average = 86%±0.9). However, the extent of *FoxP2* down-regulation in juveniles was qualitatively similar to that seen for adults [21]. (Statistical comparison is not justified since the two studies were conducted separately).

### Acute FoxP2 Down-Regulation Occurs Despite Auditory Deprivation

To determine whether or not *FoxP2* down-regulation by singing in juveniles depends on auditory feedback, 75d deafened birds were allowed to sing for 2 hours and examined *FoxP2* expression levels in area X. Similar to hearing birds, singing also decreased *FoxP2* in deafened birds relative to basal levels (3′-probe, *p*<0.005; Fig. 3). Similar results were obtained with the mid-probe or when utilizing a nidopallial region instead of outlying striatum for normalization (data not shown). No difference in the extent of down-regulation was observed between the two singing groups (3′-probe: *p* = 0.38; mid-probe: *p* = 0.52), revealing that the regulation is driven by the act of singing itself. Interestingly, unlike the hearing group in which *FoxP2* levels were correlated with the amount of singing (see above), no correlation was found for the deafened group (Spearman Rho *p* = 0.60; Fig. 4 right).

Deafened birds sang more than their hearing counterparts (75S-H vs. 75S-D in secs, range: 219–1153 vs. 271–2240, mean±SEM: 565±129 vs. 1173±209; *U* = 4.6, *p*<0.03). Thus, one concern was that the lack of correlation in the deafened group might be due to maximal down-regulation of *FoxP2* (i.e. a ‘floor’ effect) in birds who sang a lot. To gauge the likelihood of this interpretation, we considered whether removing data for the three deafened birds who sang the most (2,240, 2,013 and 1,566 secs) would reveal a correlation. The amount of singing from the remaining subset of deafened birds (range: 271–1477, mean±SEM: 845±168) was even more similar to that of hearing birds. However, this manipulation failed to reveal any correlation in the deafened birds (Spearman Rho = −0.29; *p* = 0.54, n = 7). It is important to note that a subject number (n) of 7 was sufficient to observe the correlation in the hearing group. Indeed, during these experiments, we initially collected an n of 6 in both groups and observed a significant correlation in the hearing group, but none in the deafened, as described in preliminary report (hearing vs. deaf: *p* = 0.045 vs. *p* = 0.55; Teramitsu & White, Society for Neuroscience Abstracts, 2006). To increase our confidence in these findings, we proceeded to test one additional hearing and three additional deafened birds, and obtained similar results albeit with a more significant p value in the hearing group. Since an n of 6 in the hearing group was sufficient to reveal the correlation, the lack of correlation in the subset of deafened birds (n = 6) or the full cohort (n = 10) cannot be merely attributed to a lack of power.

### Juveniles Spend More Time Singing Than Do Adult Birds

**Figure 5:**
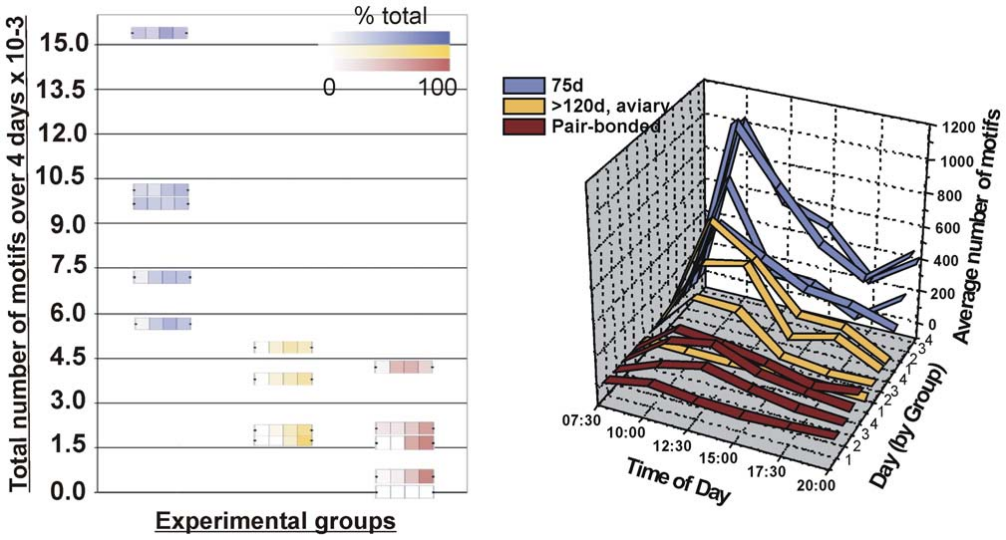
75d birds practice more than adults. Data from 75d males (n = 5) is shown in shades of purple, adult aviary males (n = 4) in gold and adult pair-bonded males (n = 5) in maroon. **Left**) The amount of song sung while in sound attenuation chambers is shown. Individual data are plotted where squares represent days 2–5 (day one was not counted to allow for acclimation) and color intensity shows percent of total motifs sung each day. Compared with older birds, 75d males tended to sing on the first recording day and sang more overall (Mean±SEM in secs: 75d = 12,958±1,731, adult aviary males = 4,494±1,042, adult pair-bonded males = 2,034±894; Kruskal-Wallis *H* = 10.7, DF = 2, *p*<0.005). **Right**) The daily pattern of singing is shown. For each group, the average number of motifs (z axis) is plotted in 2.5 hour time-blocks (x axis) across the 4 days. Each day is represented by one ribbon on the y axis and the 4 days are clustered by group.

Given that song practice lowers *FoxP2* levels in both juveniles (here, and adults [21]), we wondered whether the duration of practice differed at different ages. If so, then birds engaged in more singing at one age would presumably experience low FoxP2 levels more frequently than at the other age. To address this, we examined the daily singing patterns of males in three behavioral conditions: 75d juveniles undergoing sensorimotor learning and taken from the group aviary cage; adults (>120 days) taken from the group aviary cage, and pair-bonded adults (>120 days) taken from dedicated breeding cages. Birds were placed individually in sound attenuation chambers for 5 days while their songs were continuously recorded. Compared to both groups of older birds, 75d males tended to start singing sooner and sang more throughout the course of the experiment (Fig. 5 left panel; *p*<0.005). The greatest amount of singing occurred following light onset each day (Fig. 5, right panel).

## Discussion

Our results demonstrate that basal levels of *FoxP2* in area X of juveniles are slightly higher than those in the surrounding striatum, and only decrease acutely when birds sing. In this study, basal levels of full-length *FoxP2* remained relatively stable, exhibiting only a modest rise between 50 and 75d, consistent with the constant density of FoxP2 immunoreactive cells observed between 25-100d [16]. Our results (Fig. 2) contrast in one way with a study in which area X *FoxP2* levels were reported to rise only up to 50d, but were not statistically tested [15]. We were unable to replicate this change in pattern beyond 50d despite testing additional 75d birds using methods designed to mimic the other study and employing the same subject number (see Methods). Of note, the area X FoxP2 levels observed here in 75d non-singing birds were similar to those that we previously reported in non-singing adults (i.e. slightly higher than the surrounding striatum [21]), making it unlikely that the discrepancy is due to slight differences in the progress of song development between colonies. Differences in the probes used to detect *FoxP2* in the two studies may contribute to the different findings. The persistent expression of *FoxP2* observed here during late sensorimotor learning, the high levels of expression during human [14] and songbird embryogenesis [15], the FoxP2 immunoreactivity observed within newly generated neurons in area X [16], and the structural brain deficits in humans bearing FOXP2 mutations [41] are all consistent with a role for this molecule in the formation of certain brain regions, including the striatum.

Deafening of young birds either shortly before or at the onset of sensorimotor learning did not affect the basal expression pattern of *FoxP2* in area X at any of the three ages tested (Fig. 2) despite the expected disruption of song development [32], [40]. This suggests that basal (i.e. non-singing) *FoxP2* levels in area X are not regulated by auditory input during song development. In contrast to this relatively stable expression, when juvenile birds sang, *FoxP2* was acutely down-regulated in area X relative to the surrounding striatum (Fig. 3), similar to what we previously reported for adult birds [21]. Down-regulation occurred in both hearing and deaf birds, indicative of ‘motor-driven’ [42] gene regulation. However, the extent of down-regulation depended on the amount of singing only among hearing birds (Fig. 4), suggesting multiple layers of *FoxP2* regulation. To our knowledge, this is the first indication for an effect of audition on FoxP2 such that hearing links levels of the molecule to levels of vocal motor practice. Interestingly, transgenic mice engineered to harbor the KE family mutation in Foxp2 exhibit altered auditory brainstem responses [43]. As noted by the authors of that study, these findings suggest that humans with FOXP2 mutations should be tested for auditory function. Of note, the singing-to-*FoxP2* correlation observed here in hearing juveniles was previously observed as a trend in adults for both mRNA [21] and protein [22], but has now emerged as a significant relationship in younger birds.

The precise temporal regulation of FoxP2 that occurs only during singing, and the regional restriction of this regulation to song control nucleus area X strongly suggests that FoxP2 has a post-organizational role in learned vocalizations. Previously, we considered whether the singing driven down-regulation of *FoxP2* observed in adults [21] was related to the stereotyped nature of these songs or, alternatively, to their ongoing subtle variability. The latter possibility now seems more likely because down-regulation also occurred here when juveniles sang their more variable songs. Although the magnitude of the down-regulation appeared similar in both adults and juveniles, we found that 75d juvenile birds in our colony engaged in song practice more readily and frequently than did adults (Fig. 5). It follows that *FoxP2* levels are also more frequently low during late sensorimotor learning, when song is still changing, than in adulthood, when song is more stable. We note, however, that *FoxP2* levels were only measured at a single time point, two hours after song onset in the morning and were compared to levels after two hours of non-singing in control birds. Another difference between adults and juveniles is the stronger link between the amount of song sung and how readily *FoxP2* levels decrease at younger ages. This is evidenced by the increased strength of the correlation between these measures in 75d birds (Fig. 4) relative to adults [21], [22]. Overall, birds may tacitly ‘self-regulate’ their own *FoxP2* levels, depending on how often they engage in vocal motor practice, a relationship that may extend to other learned motor skills and other transcription factors

FoxP2 could function as a ‘plasticity gate’, either up or down, during both sensorimotor learning and adulthood. In this model, high *FoxP2* levels correspond to periods of structural growth and song stability whereas low levels open the gate for vocal variability; the more often that *FoxP2* is low, the greater opportunity for variability. Here, we refer to variability that occurs two hours after the onset of undirected singing – a more protracted timescale than the minute-to-minute changes driven by social context, e.g. [44], [45]. According to the plasticity gate hypothesis, at some point following song onset, beyond two hours, FoxP2 levels should begin to rise again in order to stabilize motor patterns, a scenario that we are currently testing. This general idea is supported by the observation that after a day of song practice, juveniles exhibit more variable songs the next morning [46]. Song practice during the day likely decreased area X FoxP2, at least transiently, in these juveniles, although the full circadian rhythm of FoxP2 levels relative to singing is not yet determined. Moreover, it may be that nighttime ‘song rehearsal’ [46] also decreases FoxP2 levels. Such variability may represent vocal motor exploration critical for improved imitation, as juveniles exhibiting the greatest morning variability end up producing the best copies of their tutors' songs. Morning increases in vocal variability decline with maturation, disappearing in adulthood [46]. Concurrently, song improves over the several week period of sensorimotor learning [26] while in adults there is a much more gradual increase in song stability across years [29]. *FoxP2* regulation could contribute to these slower changes across the lifetime of the animal, a finding supported by the constant replacement of FoxP2 immunoreactive neurons in zebra finch area X [16]. A direct prediction of this model is that songs of juveniles who sing for two hours, and thus have low FoxP2 levels, should be more variable than the songs of those same individuals when they have not sung and thus have higher FoxP2 levels, a result we have recently confirmed (see Miller et al., companion paper).

The naturally-driven down-regulation by singing observed here complements results from experimentally-induced constitutive down-regulation of FoxP2 during sensorimotor learning [47]. In the latter study, chronic down-regulation of FoxP2 using RNA interference resulted in more variable songs of 90d experimental birds relative to age-matched controls. Without the normal behaviorally-driven fluctuation in FoxP2, the songs of experimental birds were less accurate copies of the tutor. One caveat to this interpretation is that only ∼20% of area X was affected, so presumably FoxP2 levels were normally regulated in the remaining portion. FoxP2 is a transcription factor, thus the mechanism by which it exerts its function(s) is through control of downstream genes. Analysis of FOXP2 gene targets in human neural tissues reveal that a subset of these play roles in activity-based sculpting of neural connections, including during learning [48], [49]. Together, these findings suggest that while high levels of FoxP2 are important for normal development of neural structures, low levels may enable the fine-tuning of these structures during vocal motor exploration [37].
